# Probability scoring system of intravascular large B-cell lymphoma for the application of random skin biopsy: A retrospective cohort study

**DOI:** 10.1016/j.jdin.2022.09.005

**Published:** 2022-09-27

**Authors:** Mikiko Takigawa, Osamu Yamasaki, Hayato Nomura, Tomoko Miyake, Hiroyuki Yanai, Shin Morizane

**Affiliations:** a Department of Dermatology, Okayama University Graduate School of Medicine, Dentistry, and Pharmaceutical Sciences, Okayama, Japan; b Department of Diagnostic Pathology, Okayama University Hospital, Okayama, Japan

**Keywords:** Asian variant, clinical symptoms, fever of unknown origin, intravascular large B-cell lymphoma, probability scoring system, random skin biopsy, retrospective cohort study, soluble interleukin-2 receptor (sIL-2R), CI, confidence index, IVLBCL, intravascular large B-cell lymphoma, LDH, lactate dehydrogenase, PDR, positive detection rate, RSB, random skin biopsy, sIL-2R, serum soluble-interleukin-2 receptor

## Abstract

**Background:**

Intravascular large B-cell lymphoma (IVLBCL) is rare and fatal when diagnosed late in the disease course. Random skin biopsy (RSB) is useful for early diagnosis, but criteria for its application are not well established.

**Objective:**

To develop an IVLBCL-probability scoring system for stratifying patients and investigate its feasibility and capability for RSB application.

**Methods:**

A retrospective cohort of 77 consecutive patients with suspected IVLBCL who underwent RSB was included in this study. All patients were classified into 3 IVLBCL-probability groups according to the IVLBCL-probability scoring system comprising the following 4 components: general symptoms, organ-specific symptoms, serum soluble-interleukin-2 receptor levels, and serum lactate-dehydrogenase levels.

**Results:**

The high (score 7-10), intermediate (score 4-6) and low (score 1-3) IVLBCL-probability groups contained 32, 30, and 15 patients, respectively. All 5 patients with IVLBCL were stratified into the high IVLBCL probability group. Accuracies in the diagnosis of IVLBCL were 100%, 100%, and 93.8% for the low, intermediate, and high IVLBCL-probability groups. The positive detection rate in the high IVLBCL-probability group increased to 9.4% from 3.9% across all groups.

**Conclusions:**

The newly-developed IVLBCL-probability scoring system has good capability for stratification of patients and could allow limiting application of RSB for diagnosis only to high-probability groups.

## Graphical abstract

**Figure:**
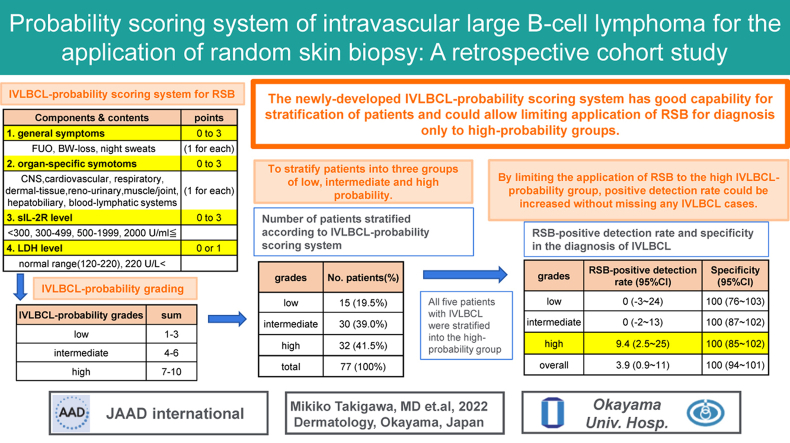

**Capsule Summary**

• The newly developed probability scoring system of intravascular large B-cell lymphoma can stratify patients into the following 3 groups: low, intermediate, and high probability.
• The application of random skin biopsy could be limited to the high-probability group without missing true positive cases, which will subsequently result in the suppression of unnecessary biopsies.

## Introduction

Intravascular large B-cell lymphoma (IVLBCL), a special subtype of diffuse large B-cell lymphoma, is a rare disease in which lymphoma cells proliferate only in the small blood vessels.^1^ IVLBCL is generally unaccompanied by lymph node lesions or mass formation. IVLBCL cells infiltrate all microvessels, subsequently resulting in a variety of clinical manifestations occurring from various organs such as the central nervous system, skin, bone marrow, lungs, and kidneys.^2^ Neurological symptoms and cutaneous manifestations are more common in the Western variant. In contrast, the Asian variant is often associated with bone marrow infiltration causing anemia, thrombocytopenia, hepatosplenomegaly, and disseminated intravascular coagulation, despite the absence of lymphadenopathy, mass formation, neurological symptoms, and skin lesions.^3^ Skin lesions of IVLBCL have a wide variety of cutaneous manifestations such as erythema, purpura, and telangiectasia.^4^

The diagnosis of IVLBCL, especially the Asian variant, is extremely difficult because of its diverse presentation and lack of lymphadenopathy. IVLBCL is fatal when diagnosed late in the disease course. Recently, random skin biopsy (RSB) has been performed before other organ biopsies, and has been reported to be useful for the early diagnosis of IVLBCL, including the Asian variant.^5^^,^^6^ However, RSB is still invasive and requires determination of biopsy sites, depth, size of skin samples, and the use of either punch or incisional biopsies.^7^ RSB positive detection rates in the diagnosis of IVLBCL have been generally low, varying from 0% to 22%,^8^, ^9^, ^10^, ^11^, ^12^, ^13^ and RSB usually yields a significant number of true negative results.

It is important to limit the application of RSB by appropriately selecting patients for RSB, particularly for the Asian variant of IVLBCL. However, clear diagnostic criteria for the application of RSB have not yet been established. Therefore, a probability scoring system for IVLBCL would help stratify patients and limit the application of RSB for patients with a high probability of IVLBCL.^14^ In this study, we aimed to investigate the feasibility of a newly developed IVLBCL-probability scoring system for the application of RSB in the diagnosis of IVLBCL.

## Subjects and methods

### Patient population

This retrospective study was approved by the ethical committee of Okayama University Hospital (No. 2201-015). Seventy-seven consecutive patients referred to our hospital and who underwent RSB between April 2011 and March 2021 were included in this study. Of the 77 patients, 38 were male and 39 were female, and the average age was 64.5 ± 53 years (range: 11-87 years). The requirement for informed consent was waived because of the retrospective nature of the study. In all 77 patients, information regarding sex, age, accompanying symptoms, blood biochemical data, histopathological findings of RSB, and final diagnosis were collected from the patients' medical records.

### Clinical and laboratory assessment

The accompanying symptoms and laboratory data were also reviewed and compared between patients with and without IVLBCL. Clinical symptoms were divided into the following 2 groups: (1) general symptoms such as fever of unknown origin, body weight loss, and night sweats, (2) organ-specific symptoms occurring from dysfunction of various organ systems, such as the central nervous system, respiratory system, cardiovascular system, dermal tissue system, renal urinary system, muscle-joint system, hepatobiliary system, and blood lymphatic system. Symptoms of the central nervous system include stroke, disturbance of consciousness, headache, and cognitive impairment. Symptoms of the respiratory system include dyspnea, hypoxemia, and respiratory failure. Symptoms of the cardiovascular system include shock and leg edema. Symptoms of the dermal tissue system include skin rash, erythema, papules, and telangiectasia. Symptoms of the renal urinary system include renal dysfunction, hematuria, and proteinuria. Symptoms of the muscle/joint system include myalgia and arthralgia. Symptoms of the hepatobiliary system include jaundice, hepatomegaly, and liver dysfunction. Symptoms of the blood lymphatic system include lymph node swelling, splenomegaly, anemia, thrombocytopenia, leukocytopenia, and pancytopenia.

For laboratory data, hematological data of white blood cells, red blood cells, platelet counts, and hemoglobin levels, and blood biochemical data, including serum lactate-dehydrogenase (LDH), C-reactive protein, serum soluble-interleukin-2 receptor (sIL-2R), and ferritin levels obtained immediately before biopsy, were reviewed.

### IVLBCL-probability scoring system

The IVLBCL-probability scoring system for the application of RSB comprises the following 4 components: general symptoms, organ-specific symptoms, sIL-2R levels, and LDH levels (Table I). In the first component, general symptoms included fever of unknown origin, body weight loss, and night sweats. One point was assigned for each symptom and a maximum of 3 points were assigned to this component. For the second component, organ-specific symptoms occurring from organ dysfunction included symptoms from the following 8 organ systems: the central nervous system, respiratory system, cardiovascular system, dermal tissue system, renal urinary system, muscle joint system, hepatobiliary system, and blood lymphatic system. One point was assigned to each organ-specific symptom and a maximum of 3 points was assigned to the second component. For the third component, the maximum of 3 points was assigned according to sIL-2R levels as follows: zero points for sIL-2R less than 300 U/ml, 1 point for that ranging from 300 to 499 U/ml, 2 points for that ranging from 500 to 1999 U/ml, and 3 points for that of 2000 U/ml or greater. For the fourth component, 1 point was given for LDH levels greater than the normal range (220 U/L).

**Table I:** IVLBCL-probability scoring system for random skin biopsy

Components	Contents	Points
1. General symptoms		0 to 3 (1 for each)
	Fever of unknown origin	+1
	Body weight loss	+1
	Night sweats	+1
2. Organ-specific symotoms		0 to 3 (1 for each)
	Central nervous system∗	+1
	Respiratory system†	+1
	Cardiovascular system‡	+1
	Dermal tissue system§	+1
	Renal urinary system‖	+1
	Muscle/joint system¶	+1
	Hepatobiliary system#	+1
	Blood lymphatic system∗∗	+1
3. sIL-2R level		0 to 3
	<300 U/ml	+0
	300-499 U/ml	+1
	500-1999 U/ml	+2
	≦2000 U/ml	+3
4. LDH level		0 or 1
	Normal range (120-220 U/L)	+0
	<220 U/L	+1

*IVLBCL*, Intravascular large B-cell lymphoma; *LDH*, lactate dehydrogenas; *sIL-2R*, soluble-interleukin-2 receptor.

∗ Central nervous system includes stroke symptoms, consciousness disturbance, headache, etc.

† Respiratory system includes dyspnea, hypoxemia, respiratory failure, etc.

‡ Cardiovascular system includes shock, leg edema, etc.

§ Dermal tissue system includes skin rash, erythema, papules, telangiectasia, etc.

‖ Renal urinary system includes renal dysfunction, hematuria,proteinuria, etc.

¶ Muscle/joint system includes myalgia, arthralgia, etc.

# Hepatobiliary system includes jaundice, hepatomegaly, liver dysfunction, etc.

∗∗ Blood lymphatic system includes lymphnode swelling, splenomegaly, anemia,thrombocytopenia,leucopenia, etc.

Points acquired for each component were summed, and the total score was graded according to the probability scale of IVLBCL as follows: low IVLBCL-probability for the total scores ranging from 1 to 3 points, intermediate IVLBCL-probability for scores ranging from 4 to 6 points, and high IVLBCL-probability for scores ranging from 7 to 10 points.

### Random skin biopsy and histopathological examination

RSB was performed by obtaining skin samples from at least 3 separate areas of the body. Punch and incisional biopsies were performed in 31 and 46 patients, respectively. Biopsy specimens were immunohistochemically stained with anti-CD20 and CD79a antibodies. Histopathological findings of intravascular lymphoma cells in biopsy specimens were considered positive for IVLBCL, whereas the absence of intravascular lymphoma cells was considered negative for IVLBCL. When RSB was negative and intravascular lymphoma cells were found in other organ biopsy samples, RSB was considered a false-negative.

### Sensitivity, specificity, accuracy, and positive detection rate

Compared to the final diagnosis obtained from the database, sensitivity, specificity, and accuracy of RSB in the diagnosis of IVLBCL with 95% confidence index (CI) were calculated for each probability group and in total. The positive detection rate (PDR) of RSB for the diagnosis of IVLBCL with a 95% CI was also calculated.

### Statistical analysis

All statistical analyses were conducted using SPSS statistics software version 26 (2019, IBM Japan). Measurable laboratory data were compared between patients with and without IVLBCL using the Mann–Whitney U test. Among-group differences were determined using the chi-squared test for the diseases among the 3 IVLBCL probability groups and clinical symptoms between patients with and without IVLBCL. Sensitivity, specificity, accuracy, and PDR of RSB in the diagnosis of IVLBCL were also compared among the low, intermediate, and high IVLBCL-probability groups using the chi-squared test. In these statistical analyses, *P*-values less than .05 were considered statistically significant.

## Results

The final diagnosis of IVLBCL was found in 5 patients (6.5%). Final diagnoses of non-IVLBCL were malignant lymphoma other than IVLBCL in 19 patients (24.7%), adult-onset Still disease in 12 (15.6%), encephalomyelitis/encephalomyelopathy in 12 (15.6%), mixed connective tissue disease including vasculitis in 9 (11.7%) patients (Table II).

**Table II:** Final diagnosis and number of patients stratified according to IVLBCL-probability scoring system

Final diagnosis	IVLBCL-probability grades			Total (%)	*P*-value
	Low∗ (%)	Intermediate† (%)	High‡ (%)		
IVLBCL	0 (0)	0 (0)	5 (15.6)	5 (6.5)	.023§
Malignant lymphoma	2 (13.3)	4 (13.3)	13 (40.6)	19 (24.7)	.023§
AOSD	0 (0)	8 (26.6)	4 (12.5)	12 (15.6)	.055
Encephalomyelitis/-pathy	7 (46.7)	5 (16.7)	0 (0)	12 (15.6)	.0002§
MCTD/vasculitis	0 (0)	7 (23.3)	2 (6.3)	9 (11.7)	.032§
TAFRO syndrome	0 (0)	0 (0)	3 (9.4)	3 (3.9)	.111
MDS·Evans syndrome	0 (0)	1 (3.3)	1 (3.1)	2 (2.6)	.779
pachymeningitis	1 (6.7)	1 (3.3)	0 (0)	2 (2.6)	.386
Multiple sclerosis	2 (13.3)	0 (0)	0 (0)	2 (2.6)	.014§
Churg-Straus syndrome	1 (6.7)	0 (0)	0 (0)	1 (1.3)	.123
subacute thyroiditis	0 (0)	1 (3.3)	0 (0)	1 (1.3)	.452
cause unspecified	2 (13.3)	3 (10)	4 (12.5)	9 (11.7)	.142
Total	15 (100)	30 (100)	32 (100)	77 (100)	

Data are shown as number of patients. Parenthesized numbers are percentages in each probability group.

*AOSD*, Adult-onset Still disease; *IVLBCL*, intravascular large B-cell lymphoma; *MCTD*, mixed connective tissue disease; *MDS*, myelodysplastic syndrome; *TAFRO*, thrombocytopenia, anasarca, fever, reticulin fibrosis, organomegaly.

∗ Low: low IVLBCL-probability (score 1-3).

† Intermediate: intermediate IVLBCL-probability (score 4-6).

‡ High: high IVLBCL-probability (score 7-10).

§ Statistically significant.

The high, intermediate, and low IVLBCL-probability groups contained 32, 30, and 15 patients, respectively (Table II). All 5 patients with IVLBCL were stratified into the high IVLBCL-probability group, where the final diagnoses of other patients were malignant lymphoma (*n* = 13), adult-onset Still disease (*n* = 4), mixed connective tissue disease (*n* = 2), TAFRO (thrombocytopenia, anasarca, fever, reticulin fibrosis, organomegaly syndrome) syndrome (*n* = 3), and others (*n* = 5). No patients with IVLBCL were included in the low and intermediate IVLBCL-probability groups. IVLBCL and malignant lymphoma were more frequent in the high IVLBCL probability group than in the low and intermediate groups (*P* = .023) (Table II).

Among general symptoms in all the patients, the most common symptoms were fever of unknown origin in 56 cases (73%), followed by weight loss in 18 cases (23%) and night sweats in 7 cases (9%) (Table III). Among organ-specific symptoms, the most common symptoms were ones from the central nervous system in 40 cases (52%), followed by the blood lymphatic system in 19 cases (25%), the respiratory system in 18 cases (23%), and the muscle/joint system in 15 cases (19%) (Table III). Body weight loss and night sweats out of the 3 general symptoms, as well as organ-specific symptoms from the respiratory system, were more frequently observed in patients with IVLBCL than in those without IVLBCL, with statistical significance.

**Table III:** Clinical symptoms that manifested in IVLBCL and non-IVLBCL patients

Symptoms		IVLBCL (*n* = 5)	Non-IVLBCL (*n* = 72)	Total (*n* = 77)	*P*-value
1. General smptoms	Fever of unknown origin	5 (100)	51 (71)	56 (73)	.157
	Body weight loss	3 (60)	15 (21)	18 (23)	.045∗
	Night sweats	3 (60)	4 (6)	7 (9)	<.0001∗
2. Organ-specific symptoms	Central nervous system	2 (40)	38 (53)	40 (52)	.580
	Respiratory system	4 (80)	14 (19)	18 (23)	.002∗
	Cardiovascular system	1 (20)	7 (10)	8 (10)	.466
	Dermal tissue system	0 (0)	13 (18)	13 (17)	.297
	Renal urinary system	1 (20)	8 (11)	9 (12)	.549
	Muscle/joint system	0 (0)	15 (21)	15 (19)	.255
	Hepatobiliary system	0 (0)	14 (19)	14 (18)	.276
	Blood lymphatic system	2 (40)	17 (24)	19 (25)	.411

Data are shown as number of patients. Parenthesized numbers are percentages in each group.

∗ Statistically significant.

**Fig 1:**
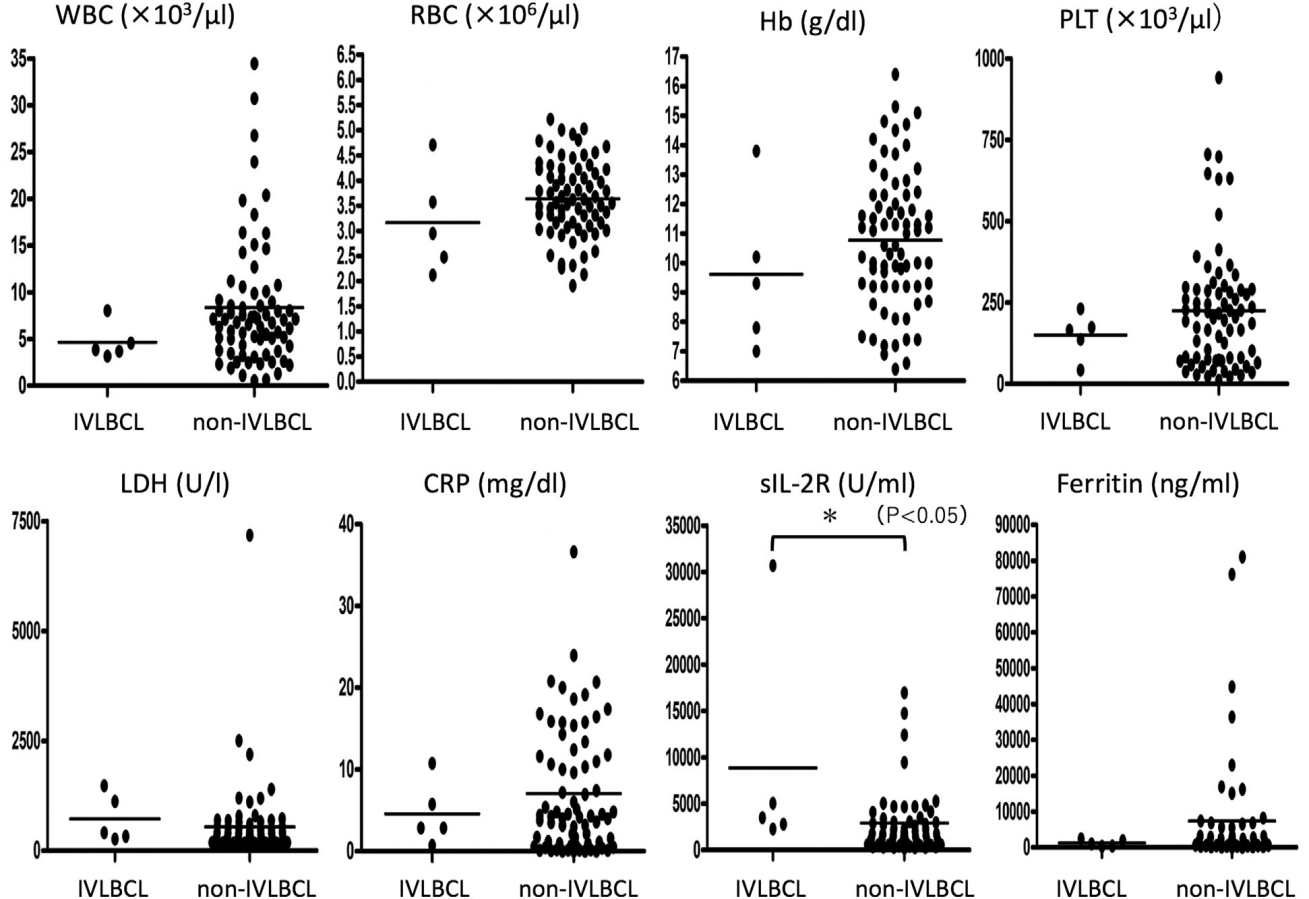
Comparison of laboratory data between IVLBCL and non-IVLBCL patients, only sIL-2R showed a significant difference (*P* = .027).

Comparison of laboratory data between the IVLBCL and non-IVLBCL cases revealed a significant difference only in sIL-2R levels, which were higher (*P* = .027) in patients with IVLBCL (median = 3488 U/ml, range: 2268-30,666 U/ml) than in patients without IVLBCL (median = 1308 U/ml, range: 190-16,900 U/ml) (Fig 1). All 5 patients with IVLBCL had sIL-2R levels greater than 2000 U/ml. The LDH level was ≥400 U/l or more in 3 of the 5 patients with IVLBCL. In addition, none of the patients with sIL-2R levels <300 U/ml and normal LDH levels had IVLBCL or malignant lymphomas.

Among the 5 patients with IVLBCL, 2 patients had positive punch biopsies, and 1 had positive incisional biopsy. In the 2 false-negative cases, punch skin biopsy was performed for 1 case and incisional biopsy was performed for the other. Overall positive detection rate of RSB was 3.9% with a 95% CI of 0.9 ∼ 11 (Table IV). According to the IVLBCL-probability grades, the high IVLBCL-probability group showed PDR of 9.4% with a 95% CI of 2.5 ∼ 25, whereas PDR was 0% in both the low and intermediate IVLBCL-probability group. Although there were no significant differences among the 3 IVLBCL-probability groups, the PDR in the high IVLBCL-probability group increased up to 9.4% from the overall PDR of 3.9%.

**Table IV:** Sensitivity, specificity, accuracy and positive detection rate of RSB in the diagnosis of IVLBCL according to the IVLBCL-probability grades

	IVLBCL-probability grades			Overall	*P*-value
	Low	Intermediate	High		
Sensitivity	NA	NA	60 (23∼88)	60 (23∼88)	NA
Specificity	100 (76∼103)	100 (87∼102)	100 (85∼102)	100 (94∼101)	NA
Accuracy	100 (76∼103)	100 (87∼102)	93.8 (79∼99)	97.4 (90∼99)	.236
Positive detection rate	0 (−3∼24)	0 (−2∼13)	9.4 (2.5∼25)	3.9 (0.9∼11)	.111

Data are shown as percentage. Parenthesized numbers are 95% CI.

*CI*, Confidence index; *IVLBCL*, intravascular large B-cell lymphoma; *NA*, not applicable.

In the 2 patients with false-negative findings of RSB, organ biopsy specimens other than skin revealed positive histopathological findings for IVLBCL cells as follows: biopsy specimens from the lung in 2 patients with IVLBCL, and from the bone marrow and kidney in one patient each. RSB showed no false-positive findings. The overall sensitivity, specificity, and accuracy of RSB for the diagnosis of IVLBCL were 60%, 100%, and 97.4%, respectively (Table IV). Specificities were 100% in all 3 IVLBCL-probability groups. Sensitivities could not be calculated in either the low or intermediate IVLBCL-probability group because no IVLBCL patients were included in those groups, whereas, sensitivity was 60% in the high IVLBCL-probability group. Accuracies were 100%, 100%, and 93.8% for the low, intermediate, and high IVLBCL-probability groups without any statistical significance, respectively.

## Discussion

This study indicated that the newly developed IVLBCL-probability scoring system performed well in stratifying patients suspected of having IVLBCL. All 5 patients with IVLBCL were classified into the high-probability group. None of the patients with IVLBCL were included in the low- or intermediate-probability groups. The overall positive detection rate was only 3.9% and as low as previously reported.^9^^,^^10^^,^^12^^,^^14^ RSB resulted in true-negative results for most patients in this retrospective cohort. In clinical settings, this newly developed IVLBCL-probability scoring system could be a method to increase positive detection rates by limiting the application of RSB to the high IVLBCL-probability group without missing any IVLBCL cases.

In the development of this IVLBCL-probability scoring system for the application of RSB, clinical symptoms were considered in addition to blood biochemical data of sIL-2 and LDH. To date, laboratory data have been suggested as a benchmark for determining the indications for RSB.^2^^,^^13^, ^14^, ^15^ Sumi-Mizuno stated that the indication for RSB was sIL-2R ≥ 2000 U/ml and that RSB was not recommended when sIL-2R was less than 500 U/ml, and LDH was normal.^16^ In the present study, all the patients with IVLBCL had sIL-2R levels >2000 U/ml, while 23 (31.9%) out of the 72 patients without IVLBCL also had sIL-2R levels >2000 U/ml. Thus, sIL-2R and LDH were employed in the IVLBCL-probability scoring system (Table I). The sIL-2R scale was subsequently set at 300, 500, and 2000 U/ml. The LDH threshold was also set at the upper limit of the normal range of 220 U/L.

However, laboratory data alone would not always be perfect in narrowing down the suspicion spectrum of IVLBCL.^14^^,^^16^ Therefore, a combination of clinical symptoms and blood biochemical data was implemented in the IVLBCL-probability scoring system. Both general and organ-specific symptoms were incorporated into the probability scoring system. The first component of general symptoms included the 3 major common symptoms that manifest in malignant lymphoma as well as IVLBCL. The second component of organ-specific symptoms included 8 organ systems, which patients with IVLBCL commonly present with.^1^^,^^2^ Thus, this newly-developed IVLBCL-probability scoring system was successful in stratifying patients suspected to have IVLBCL according to the probability grade of IVLBCL.

RSB is convenient and useful in the early diagnosis of IVLBCL because the skin is easily accessible for biopsy as compared to other organs.^5^^,^^9^ However, RSB has very low positive detection rates, which shows that a considerable number of true-negative results are included in patients who undergo RSB.^16^ Therefore, it is important to decrease true negative results by appropriately selecting patients for RSB because RSB is still invasive for patients even when the procedure is performed with either punch or incisional biopsy.^9^ The IVLBCL-probability scoring system performed well in stratifying patients suspected of having IVLBCL and also should encourage RSB to be done in patients with high IVLBCL-probability. Consequently, it would be capable of helping reduce RSB in low and/or intermediate IVLBCL-probability groups and to limit applications of RSB to the high IVLBCL-probability group without missing IVLBCL cases. In addition, even when RSB shows negative results, this IVLBCL-probability scoring system could help prioritize the high IVLBCL-probability group to proceed to other organ biopsies.

Our study had some limitations. First, the sample size might not be sufficiently large for statistical significance. However, the preliminary results of this retrospective cohort study demonstrated the feasibility and capability of the newly developed IVLBCL-probability scoring system in the stratification of patients suspected of having IVLBCL, and in narrowing the application of RSB to the high IVLBCL-probability group. Further investigation in a large-scale study, such as a multi-institutional trial, should be conducted to establish the usefulness of the IVLBCL probability scoring system. Second, there were differences in the biopsy techniques, such as punch or incisional biopsy. However, no clear differences have been noted in the positive detection rates of RSB between punch and incisional biopsies,^8^^,^^9^^,^^11^, ^12^, ^13^ as was also shown in this study. Third, the sampling sites for RSB were not always taken from skin lesions. The presence of skin lesions is not an absolute prerequisite for obtaining positive skin specimens.^14^ In this study, the positive detection of IVLBCL by RSB was also independent of the sample sites and skin biopsy techniques.

In conclusion, the newly-developed IVLBCL-probability scoring system could stratify patients suspected of having IVLBCL for RSB and limit the application of RSB to the high-probability group, which would subsequently result in an increase in the positive detection rate in the diagnosis of IVLBCL.

## Conflicts of interest

None disclosed.
